# The complex relationship between vitamin D and kidney stones: balance, risks, and prevention strategies

**DOI:** 10.3389/fnut.2024.1435403

**Published:** 2024-09-13

**Authors:** Fan Zhang, Wenjian Li

**Affiliations:** ^1^Department of Endocrinology, Changzhou Third People’s Hospital, Changzhou Medical Center, Nanjing Medical University, Changzhou, China; ^2^Department of Clinical Nutrition, Changzhou Third People’s Hospital, Changzhou Medical Center, Nanjing Medical University, Changzhou, China; ^3^Department of Urology, Changzhou Third People’s Hospital, Changzhou Medical Center, Nanjing Medical University, Changzhou, China

**Keywords:** vitamin D, kidney stones, calcium and phosphorus metabolism, personalized medicine, lifestyle intervention

## Abstract

The association between vitamin D and kidney stones is characterized by a remarkable multi-dimensional complexity involving numerous physiological and metabolic pathways. Vitamin D is pivotal in maintaining calcium-phosphorus metabolic homeostasis and bone health. However, fluctuations in its intake, whether excessive or insufficient, May potentially increase the risk of kidney stones. Vitamin D exerts its influence on kidney stone formation indirectly by increasing the efficiency of intestinal calcium absorption and regulating renal calcium excretion. Moreover, there is a robust correlation between various states of vitamin D, particularly its active form, 1,25-dihydroxyvitamin D, and the development of numerous kidney stones. This finding underscores the necessity of individualized medical treatment in vitamin D supplementation and kidney stone prevention. When developing treatment strategies, it is essential to consider the patient’s genetic background, lifestyle, environmental factors, and overall health. To prevent the formation of kidney stones, it is recommended that patients adopt a comprehensive approach, which May include measures such as moderate sun exposure, dietary modification, moderate exercise, and weight management. These preventive measures are designed to maintain healthy calcium and phosphorus metabolism and reduce kidney stone formation risk. Future studies should aim to elucidate the detailed mechanisms of vitamin D metabolism, individual differences, and the role of genes in this process. Furthermore, the role of lifestyle interventions in preventing kidney stones requires greater attention. Moreover, the implementation of large-scale, long-term prospective studies and randomized controlled trials will facilitate the assessment of the actual effects of diverse vitamin D supplementation strategies, thereby providing a robust scientific foundation for advancing more precise prevention strategies and clinical guidelines.

## 1 Introduction

Vitamin D, a widely recognized nutrient, has recently attracted significant attention from the scientific community and the general public. Its physiological role in the human body is far-reaching and extensive. It is directly involved in calcium and phosphorus metabolism and the maintenance of bone health and plays a vital role in regulating immune function (1, 2). The biological effects of vitamin D extend beyond those related to bone health, as it has been shown to influence several physiological processes, thereby underscoring its indispensable value. Conversely, kidney stones, a prevalent affliction of the urinary system, are precipitated by many factors, including dietary habits, lifestyle, and genetic predispositions (3–6). Although the precise mechanism of kidney stone formation remains to be elucidated, it is widely accepted that the deposition of calcium, uric acid, and oxalic acid is a primary causative factor (7–9).

An in-depth investigation of the relationship between vitamin D and kidney stones is of critical importance for a comprehensive understanding of the physiologic functions of vitamin D and the pathogenesis of kidney stones. A deficiency in vitamin D has been demonstrated to potentially elevate the likelihood of developing kidney stones (10–12). Consequently, administering an appropriate vitamin D dosage to individuals exhibiting deficiencies May prove a productive method for mitigating the risk of kidney stones. Nevertheless, there is no consensus among the academic community regarding the precise relationship between vitamin D intake and the risk of developing kidney stones. Some studies indicate that vitamin D supplementation May result in elevated blood calcium levels, subsequently increasing urinary calcium excretion and ultimately elevating the risk of kidney stones (13–16). However, other studies have reached the opposite conclusion, indicating that although vitamin D supplementation May result in alterations in calcium metabolism and an increased risk of hypercalcemia and hypercalciuria, it does not increase the risk of kidney stones (17, 18). Some studies have concluded that vitamin D supplementation does not significantly affect serum calcium concentration and urinary calcium excretion (19, 20). It is evident that the relationship between vitamin D and kidney stones is complex and requires further investigation. Consequently, a comprehensive investigation into the relationship between vitamin D and kidney stones is paramount. This helps us understand the physiological function of vitamin D more comprehensively and provides new ideas and methods for preventing and treating kidney stones. This article aims to review the relevant studies conducted in recent years comprehensively. This review is intended to serve as a valuable reference for researchers and clinical practitioners in related fields and facilitate the advancement of academic knowledge and clinical applications in this field.

## 2 Physiological functions of vitamin D

### 2.1 Sources and metabolic pathways of vitamin D

Vitamin D is a crucial fat-soluble vitamin that maintains bodily health. It has two primary forms: vitamin D2 (ergocalciferol) and vitamin D3 (cholecalciferol) (21). Vitamin D2 is primarily derived from plant sources, while the skin synthesizes vitamin D3 in response to sunlight. The synthesis of vitamin D3 begins with 7-dehydrocholesterol (7-DHC) in the skin, which is converted to cholecalciferol in the presence of ultraviolet B (UVB) radiation (22). In addition to dermal synthesis, vitamin D can be ingested through food, particularly oily fish (e.g., salmon and mackerel), fortified dairy products, egg yolks, and certain mushrooms high in the vitamin (23–25).

**Figure 1:**
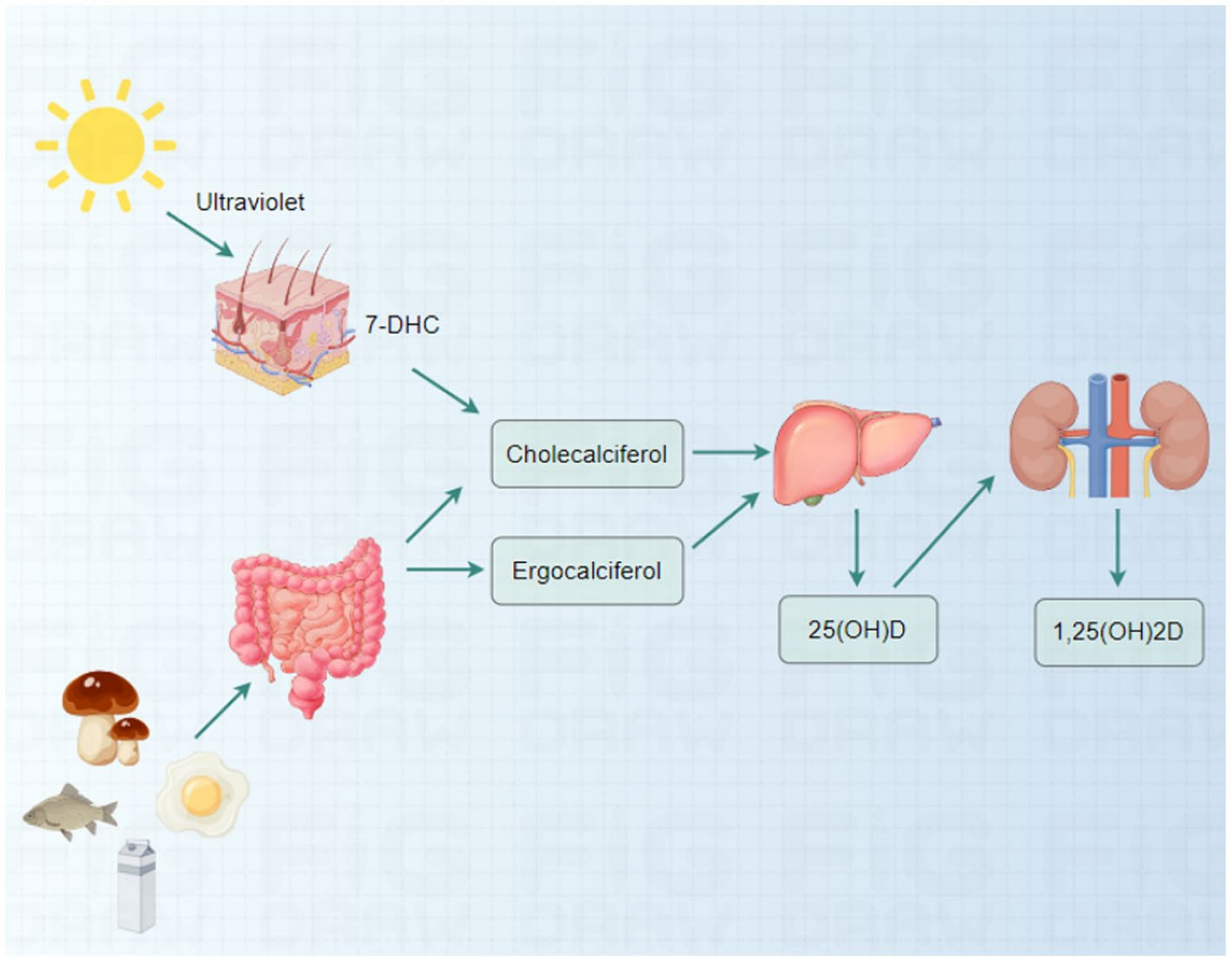
Sources and metabolic pathways of vitamin D.

The activation of vitamin D in the body necessitates a two-step conversion process. First, vitamin D is converted to 25-hydroxyvitamin D (25(OH)D), its primary circulating form in the liver (26). Subsequently, in the kidneys, 25(OH)D is further converted to 1,25-dihydroxyvitamin D (1,25(OH)_2_D), the active form of vitamin D responsible for regulating calcium and phosphorus metabolism (Figure 1) (27–29).

### 2.2 Role of vitamin D in calcium and phosphorus metabolism

Vitamin D plays a crucial role in maintaining the body’s equilibrium of calcium and phosphorus. It Markedly enhances the bioavailability of these two essential minerals by stimulating the active intestinal absorption processes of calcium and phosphorus (30, 31). Specifically, vitamin D binds to the vitamin D receptor (VDR) in intestinal cells, activating several physiological responses. One significant consequence is an enhancement in the efficacy of calcium transport across the gastrointestinal epithelium via the transient receptor potential vanilloid 6 (TRPV6) transporter protein, which optimizes the intestinal absorption of calcium (32). Vitamin D sufficiency induces intestinal cells to synthesize calcium-binding proteins, which bind specifically to calcium ions, enhancing the intestinal affinity for calcium ions and thus facilitating calcium absorption (33, 34). Furthermore, vitamin D benefits phosphorus absorption, thereby ensuring the effective utilization of phosphorus in the body.

Vitamin D plays a pivotal role in regulating hormone secretion, thereby maintaining the blood’s equilibrium of calcium and phosphorus concentrations. This mechanism is primarily achieved through the influence of vitamin D on the secretion of parathyroid hormone (PTH) and fibroblast growth factor 23 (FGF23) (35, 36). In the metabolic milieu of the kidney, the interaction between vitamin D, FGF23, and PTH is essential for maintaining homeostasis of calcium and phosphorus metabolism (37). Specifically, when vitamin D activity is enhanced, it Decreases the level of PTH. This, in turn, Decreases renal reabsorption of calcium and phosphorus, leading to increased urinary excretion of calcium and phosphorus. Conversely, elevated vitamin D activity stimulates the expression of FGF23, which, by inhibiting the activity of 1α-hydroxylase (CYP27B1) and activating 24-hydroxylase (CYP24A1), a pivotal enzyme in the degradation of vitamin D, in turn reduces the production of active vitamin D (1,25(OH)_2_D) (38). In contrast, PTH plays a distinct role in maintaining calcium-phosphorus balance. When the concentration of calcium in the bloodstream Declines, the secretion of PTH is initiated, which, in turn, increases the reabsorption of calcium by the kidneys (38). Concurrently, PTH stimulates the production of 1,25(OH)_2_D in the kidneys by increasing the activity of 1α-hydroxylase and Decreasing the activity of 24-hydroxylase, thereby enhancing intestinal calcium absorption and elevating the calcium concentration in the blood (39). In the presence of elevated blood calcium levels, vitamin D stimulates the production of FGF23, which, by reducing the reabsorption of phosphorus by the kidneys, helps to lower blood phosphorus concentrations (35). This, in turn, prevents the onset of physiological disturbances that high blood calcium levels May trigger. This intricate and precisely calibrated regulatory network ensures the maintenance of calcium and phosphorus metabolism within the body, which is crucial for preserving bone health and sustaining other physiological processes.

The impact of vitamin D on the process of bone remodeling is considerable. Skeletal remodeling is a complex and dynamic process involving a sophisticated interaction between osteoblasts, primarily responsible for bone formation, and osteoclasts, mainly responsible for bone resorption (40). Vitamin D plays a pivotal regulatory role in this process, influencing and optimizing the functional state of both types of cells involved in bone renewal and repair. Vitamin D significantly enhances the activity of osteoblasts, which facilitates the synthesis of bone matrix, thereby reinforcing the bone structure. Concurrently, vitamin D also inhibits osteoclast activity, reducing bone loss and maintaining bone stability and integrity (37, 41–43). This two-way regulation mechanism of vitamin D enables the dynamic balance of the bone remodeling process to be better kept, thus effectively preventing osteoporosis and other bone-related diseases.

### 2.3 Distribution and function of vitamin D receptors in the urinary system

The VDR is a nuclear receptor widely distributed in several body tissues, including the kidneys, urinary tract, and bladder of the urinary system (44). The expression and activity of the VDR are critical to the health and function of the urinary system.

The VDR plays a pivotal role in the regulation of renal electrolyte homeostasis. The VDR is expressed in renal tubular epithelial cells, and vitamin D is involved in the renal reabsorption and excretion of calcium and phosphorus by binding to it (45, 46). This regulatory mechanism is essential for maintaining calcium-phosphorus balance within the body and preventing disorders of mineral metabolism. Vitamin D affects the acid–base regulatory mechanism of the kidneys through the VDR, thereby assisting in maintaining the acid–base balance of urine (47, 48). This action is of significant importance in preventing urate and other acid deposition within the urinary tract, thereby preventing the formation of kidney stones. Moreover, VDR May play a role in the indirect regulation of renal electrolyte balance, extending beyond calcium and phosphorus, through its influence on the renin-angiotensin-aldosterone system (RAAS) activity (49). Vitamin D plays a regulatory role in the reabsorption of sodium, potassium, and other electrolytes in the kidneys, which in turn affects the production and excretion of urine. Vitamin D can potentially exert protective effects in chronic kidney disease (CKD) patients. The activation of the VDR has been demonstrated to slow the progression of kidney disease, with mechanisms that May be related to anti-inflammatory, anti-fibrotic, and immunomodulatory functions (50–53). The expression of the VDR in bladder tissues May be linked to smooth muscle function and the urinary storage capacity of the bladder (54). However, research in this area has been relatively limited. The immunomodulatory role of vitamin D is also essential in the urinary system, where it May prevent infection and inflammation by modulating local immune responses (12, 55).

## 3 Relationship between vitamin D and kidney stones

### 3.1 Mechanism of vitamin D in kidney stone formation

Vitamin D, particularly its active form, 1,25(OH)_2_D, plays a complex and multifaceted role in forming kidney stones. Specifically, vitamin D facilitates intestinal calcium absorption by binding to the VDR on intestinal cells (32, 34, 56, 57). This physiological process May result in elevated blood calcium levels, leading to hypercalcemia. In a hypercalcemic state, the inhibition of calcium transport May outweigh the inhibition of sodium and calcium absorption by medullary collaterals, ultimately resulting in increased urinary calcium excretion (58). Furthermore, elevated 1,25(OH)_2_D circulating levels May contribute to an additional increase in urinary calcium excretion (59, 60). Many studies have demonstrated that vitamin D intake increases urinary calcium excretion (18, 61, 62). For patients with hypercalciuria, a population at high risk for stone formation, the increased calcium excretion due to vitamin D intake will further exacerbate their risk of stone formation. This is because patients with hypercalciuria are more susceptible to stone formation. Even minor elevations in urinary calcium can precipitate a notable increase in calcium oxalate supersaturation.

1,25(OH)_2_D also regulates the expression of calcium-sensing receptors (CaSR) and calcium-binding proteins in the kidney and other tissues (45, 63–65). These proteins play a pivotal role in intracellular calcium transport and excretion. The CaSR can effectively inhibit paracellular calcium transport by up-regulating claudin-14 expression, reducing renal tubular permeability to calcium ions and thus maintaining stable intracellular calcium concentrations (64, 65). Transient receptor potential cation channel subfamily C, member 3 (TRPC3) is expressed in the proximal tubule (PT) and plays a role in the transcellular calcium reabsorption process at this site through CaSR activation (66). It is noteworthy that TRPC3 knockout mice exhibited hypercalciuria and microcalcification, demonstrating the protective role of TRPC3 in preventing kidney stone formation (66). Furthermore, proximal tubule cells are particularly susceptible to oxidative damage from excess reactive oxygen species (ROS) (67, 68). In a mouse model of TRPC3 ablation, increased hypercalciuria resulted in heightened oxidative stress, precipitating PT cell injury and ultimately forming mixed stones. This finding further confirms the critical role of TRPC3 in the occurrence and development of kidney stones (69). Furthermore, it has been demonstrated that TRPC3-like proteins are involved in the vital process of capacitative cation entry induced by 1α,25-dihydroxyvitamin D3 under conditions of ROS (70). Consequently, vitamin D deficiency or excess May disrupt the typical expression of these pivotal proteins, resulting in an imbalance in calcium reabsorption and excretion and, ultimately, an elevated risk of kidney stone formation.

The etiology of kidney stones is complex, with calcium oxalate stones accounting for more than 80% of cases (71). The formation of calcium oxalate stones is influenced by a combination of factors, with hypercalciuria and hyperoxaluria representing two major risk factors significantly elevating the risk of stones. In-depth studies have revealed that oxalate secretion in renal tubules is mediated through the SLC26A6 protein in the solute linkage carrier 26 gene family (72). Notably, studies in mouse models have demonstrated a significant increase in urinary oxalate concentration and stone formation rate in the presence of upregulated SLC26A6 expression (73). Vitamin D is pivotal in oxalate metabolism and contributes to kidney stone formation. The equilibrium between the absorption and excretion of oxalate, the primary constituent of calcium stones, is paramount in preventing stone formation (74). It has been demonstrated that vitamin D supplementation May accelerate the formation of Randall’s plaque, which represents the initiating step in calcium oxalate stone formation (75). Furthermore, vitamin D supplementation May enhance oxalate absorption in the gut, which can result in the development or worsening of hyperoxaluria, thereby increasing the relative saturation of calcium oxalate (76, 77). Both of these conditions can potentially promote the formation of oxalate stones.

**Figure 2:**
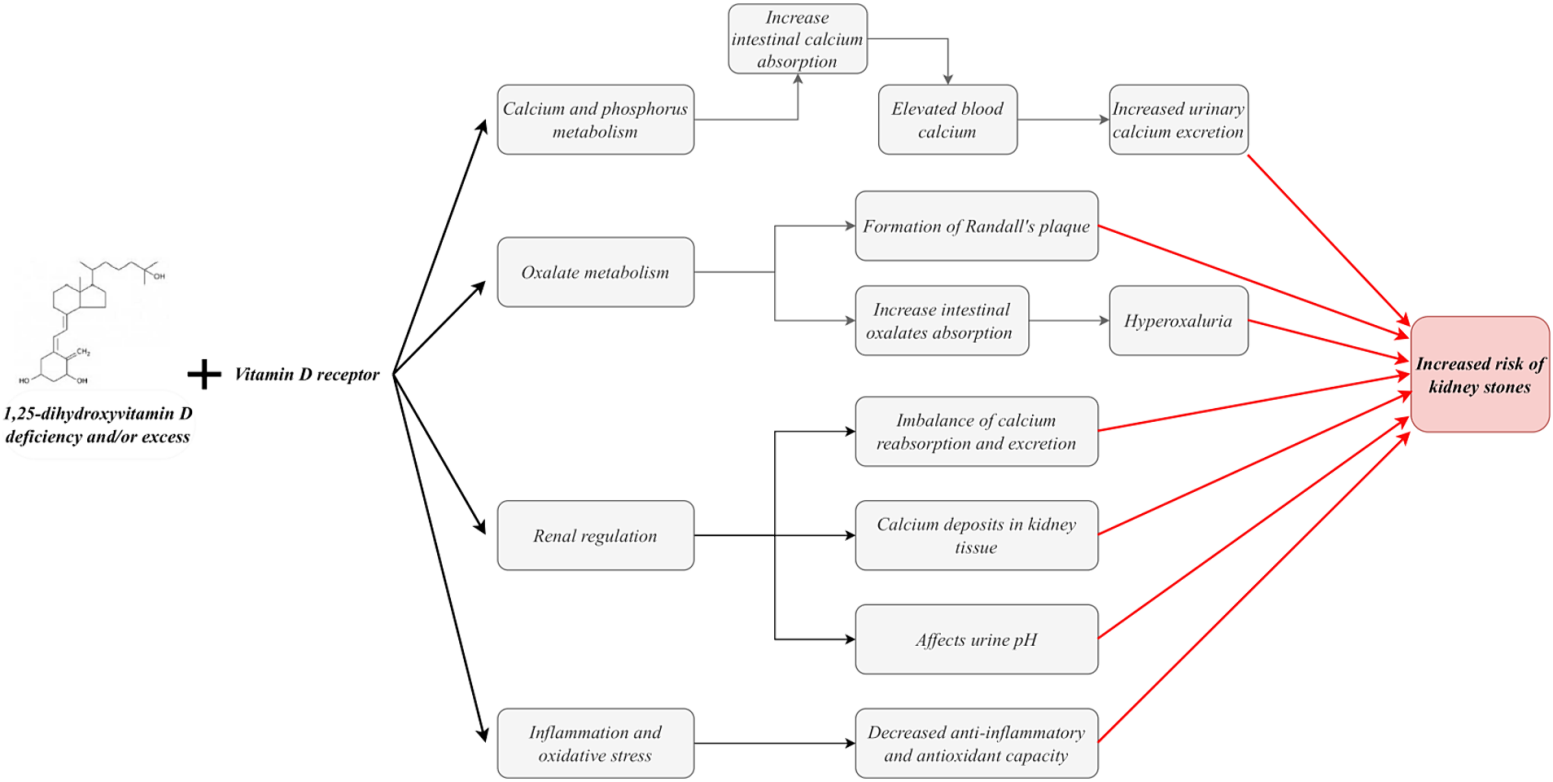
Mechanism of vitamin D in kidney stone formation.

Vitamin D plays a pivotal role in regulating calcium deposition in renal tissues. This is achieved primarily through the modulation of the activity of renal calcification inhibitory factors, including receptor activator of nuclear factor kappa-B ligand (RANKL) and osteoprotegerin (OPG) (78, 79). The equilibrium of these factors is paramount for the average deposition of calcium salts in the kidney. When imbalanced, it May result in the abnormal deposition of calcium salts in the kidney, which induces stone formation. Furthermore, vitamin D is implicated in the renal acid–base regulatory mechanism, which modulates the solubility of calcium and oxalate by altering the pH of the urine. Changes in urinary pH significantly impact stone formation, directly affecting the solubility of these minerals in the urine (80). Notably, pathological processes such as inflammation, oxidative stress, and angiogenesis have also been strongly associated with kidney stone formation. Vitamin D’s anti-inflammatory, antioxidant, and anti-angiogenic properties are essential. These bioactivities mitigate the damage to renal tissue caused by inflammation and oxidative stress and May also play a preventive role by directly intervening in the stone formation process (Figure 2) (12, 81).

Hypercalciuria and calcium kidney stones represent significant risk factors for CKD. The regulatory role of vitamin D in these pathological processes is highly complex. While the exact mechanism has not yet been fully elucidated, it is known to prevent the development of hypercalciuria and calcium kidney stones primarily by regulating blood calcium levels and influencing urinary calcium excretion. Reducing blood calcium levels increases PTH secretion, stimulating the kidneys to produce more significant quantities of 1,25(OH)₂D. This response facilitates the absorption of calcium in the intestines and the release of calcium from bones, thereby maintaining stable blood calcium levels. Conversely, vitamin D metabolism is inhibited when blood calcium levels are elevated, reducing calcium absorption and preventing hypercalcemia. It is important to note that although vitamin D does not directly regulate urinary calcium excretion, it indirectly affects this process by influencing PTH secretion and renal VDR activity. In typical circumstances, vitamin D and PTH function in concert to ensure the equilibrium of calcium levels in the blood and urine. It is also important to note that prolonged excessive vitamin D intake May result in hypercalcemia and hypercalciuria. Consequently, the regulation of vitamin D requires particular caution for patients presenting with hypercalciuria and calcium kidney stones. It is crucial to maintain reasonable control of vitamin D intake and to conduct close monitoring of blood and urine calcium levels to prevent the development of CKD.

The role of vitamin D becomes particularly complex and essential in the context of patients with CKD. CKD is often accompanied by disturbances in vitamin D metabolism, and vitamin D deficiency is commonly observed in CKD and its disease progression. A comprehensive analysis of 367 pre-dialysis patients revealed that over 80% exhibited vitamin D deficiency. This deficiency reflects the significant impact of Decreased renal function on the anabolic and catabolic phases of vitamin D metabolism. Further, it disturbs the calcium and phosphorus homeostasis, significantly increasing the risk of kidney stone formation (82).

### 3.2 Potential link between vitamin D deficiency and kidney stones

Vitamin D deficiency is typically diagnosed when the concentration of 25(OH)D is less than 20 ng/mL (44, 83, 84). A literature review reveals a significantly higher prevalence of vitamin D deficiency in individuals with urolithiasis than those without (11). Vitamin D deficiency May induce secondary hyperparathyroidism, a compensatory physiological response by the body to maintain homeostasis of blood calcium levels. This is achieved specifically through increased secretion of PTH (12, 83). Elevated levels of PTH further promote the release of calcium from bone into the bloodstream and inhibit renal reabsorption of calcium, leading to increased urinary calcium excretion. These physiologic changes May elevate the risk of specific types of kidney stones (85). Furthermore, vitamin D deficiency May exacerbate the risk of kidney stone formation by inducing oxidative stress and overexpression of inflammatory mediators in renal tissue (12). The impact of vitamin D deficiency on renal reabsorption function, encompassing the reabsorption of calcium and phosphorus, should also be considered. Despite the lack of complete elucidation, the potential association between vitamin D deficiency and renal function with renal stone formation merits further comprehensive investigation.

### 3.3 Correlation between vitamin D supplementation and the risk of kidney stones

Vitamin D is pivotal in maintaining bone health and regulating calcium and phosphorus metabolism. The normal range for vitamin D is generally considered 20–50 ng/mL (50–125 nmol/L) (86). However, levels above 100 ng/mL May indicate an overdose, while levels above 150 ng/mL are indicative of vitamin D toxicity. At present, there is no standardized dose of vitamin D supplementation. The usual recommended daily supplementation dose is 400 to 800  international units (IU) (87). However, daily supplementation of approximately 2,000 IU of vitamin D May be necessary to achieve serum 25(OH)D concentrations of ≥30 ng/mL in most of the population (88). The tolerable maximum daily vitamin D intake is typically 4,000 IU/day. It is important to note that supplementation of more than 10,000 IU of vitamin D daily May have toxic effects on the general population (89). The relationship between vitamin D supplementation dose and vitamin D concentration is not linear and is influenced by various factors, including age, gender, health status, and sun exposure.

Academic researchers have no consensus regarding the relationship between vitamin D supplementation and kidney stone risk. Some studies have substantiated the correlation between vitamin D supplementation and an increased risk of kidney stones. In humans and rats, vitamin D supplementation has been demonstrated to result in the development of hypercalciuria, renal calcification, and/or kidney stone formation (17, 90). Several meta-analyses have indicated that vitamin D supplementation increases the risk of kidney stones (13–15). A 3-year, double-blind, randomized controlled trial demonstrated that in healthy adults, vitamin D3 supplementation (400, 4,000, or 10,000 IU per day) resulted in hypercalciuria in 23% of participants, with a higher prevalence observed in the high-dose group (91). Another study of patients with recurrent calcium kidney stones and concomitant vitamin D deficiency revealed that serum 25(OH)D and 24-h urinary calcium levels exhibited a Marked elevation following 8–12 weeks of treatment with 50,000 IU of vitamin D per week (18). In contrast, a meta-analysis indicated that prolonged (≥24 weeks) vitamin D supplementation was associated with an elevated risk of hypercalcemia and hypercalciuria, with no discernible correlation between dosage and risk (17). A longitudinal prospective study also observed that total vitamin D intake was associated with an elevated risk of kidney stones in a cohort of female nurses aged 25–42 (92). Similarly, supplemental vitamin D intake yielded comparable outcomes (92). A randomized clinical trial conducted by the Women’s Health Initiative demonstrated that postmenopausal women who received daily oral vitamin D3 (400 IU) plus calcium (1,000 mg) supplementation exhibited a 17% higher incidence of urinary tract stones compared to the placebo group after a 7-year follow-up period (93, 94). Furthermore, a 4-year, double-blind, placebo-controlled, randomized clinical trial revealed that in healthy postmenopausal women aged 55 years or older, the incidence of both kidney stones and elevated serum calcium levels was significantly higher in the treatment group (receiving vitamin D3 2000 IU/day and calcium 1,500 mg/day) than in the placebo group (95). A Mendelian randomization study also corroborated the correlation between chronically elevated circulating 25-hydroxyvitamin D levels and elevated blood calcium levels, which were associated with an increased risk of kidney stones (16). Vitamin D toxicity can result in hypercalciuria and the formation of kidney stones (96). Polymorphisms in the VDR gene have been demonstrated to affect calcium metabolism, which has been identified as a trigger for the formation of urinary stones (97–99). In patients with recurrent renal stones, vitamin D3 is regarded as a pivotal hormone in the pathogenesis, which May elevate the risk of kidney stones by Augmenting urinary excretion of calcium and phosphorus (60). Vitamin D administration has demonstrated a favorable impact on mitigating the severity of COVID-19, thereby warranting its recommendation as an adjunctive therapeutic option for managing the disease (100, 101). However, it is imperative to consider the potential risk of hypercalciuria and the development of renal stones when utilizing vitamin D in treating patients with COVID-19 (102).

Nevertheless, some studies adopt an alternative perspective. The findings of these studies indicate that, although vitamin D supplementation results in alterations in calcium metabolism and an increased risk of hypercalcemia and hypercalciuria, it does not consequently elevate the risk of kidney stones (17, 18). Other studies have demonstrated that vitamin D supplementation does not impact serum calcium concentrations or urinary calcium excretion (19, 20, 103). For instance, an analysis of individuals with vitamin D deficiency and stone formation demonstrated that 50,000 IU of vitamin D for 8 weeks did not result in elevated urinary calcium levels (103). Another randomized clinical trial examined the effects of low- and high-dose vitamin D supplementation (receiving 1,000 IU of vitamin D per day or 50,000 IU of vitamin D per week for 6 weeks) in vitamin D-deficient stone formers. The results demonstrated that vitamin D supplementation did not increase urinary calcium excretion or calcium oxalate supersaturation (20). Furthermore, a randomized, placebo-controlled, double-blind clinical trial showed that high-dose vitamin D therapy (100,000 IU/month) did not elevate the risk of stone formation or hypercalcemia in the general population (19).

The available literature indicates that moderate supplementation with vitamin D does not typically result in the formation of kidney stones. Vitamin D supplements within the recommended dosage range (less than 4,000 IU per day) do not generally result in imbalances in calcium metabolism or an increased risk of kidney stones. However, it is possible that long-term use of vitamin D May increase the risk of kidney stones, especially when combined with calcium supplementation. This is because excessive vitamin D intake can stimulate an excessive absorption of calcium from the intestines, resulting in elevated calcium concentrations in the blood. When the calcium concentration in the blood is elevated, the excess calcium is excreted in the urine, thereby increasing the calcium concentration in the urine. Furthermore, high-calcium urine is a significant contributing factor to kidney stone formation. When stone-forming substances such as oxalic acid or phosphate are also elevated in the urine, the likelihood of forming calcium oxalate or calcium phosphate stones is increased.

## 4 Prevention and treatment strategies

### 4.1 Kidney stone prophylaxis for vitamin D status

Maintaining a healthy vitamin D status is essential to prevent kidney stone formation. Sun exposure is an effective method for the natural synthesis of vitamin D3. It is recommended that individuals engage in moderate sun exposure during the hours of the day when the sun is at its highest point in the sky to facilitate the synthesis of vitamin D within the body. Concurrently, the diet should include foods rich in vitamin D, such as oily fish, cod liver oil, fortified dairy products, and egg yolks (104–106). Furthermore, ensuring an adequate calcium intake can also help reduce oxalate absorption, reducing the risk of calcium oxalate stone formation.

Vitamin D supplements May be considered viable for individuals who cannot obtain adequate amounts of vitamin D through natural sunlight exposure or daily dietary intake. Previous studies have demonstrated that a weekly intake of 30,000 IU of vitamin D supplementation is an effective means of assisting individuals with vitamin D deficiency to achieve standardized vitamin D levels (>30 ng/mL). Further studies have demonstrated that administering the same vitamin D supplements twice weekly for 5 weeks represents a rapid, productive, and secure treatment option for vitamin D deficiency (107). It is crucial to acknowledge that obese patients typically require a dosage of vitamin D supplementation that is two to three times greater than that required by individuals with average body weight when treating vitamin D deficiency (108). This is because obesity May result in a reduction in the bioavailability of vitamin D within the body. Physicians should adhere to clinical guidelines based on the latest research evidence when considering vitamin D supplements for patients with kidney stones (83). Several factors must be considered when making this Decision, including the patient’s vitamin D status, the chemical composition of the stones, kidney function, and overall health. While supplementation is necessary for patients with vitamin D deficiency, it is essential to avoid excessive intake to minimize the risk of hypercalciuria and kidney stones.

Vitamin D supplements May be necessary in certain specific situations, such as in patients with osteoporosis, as they help improve bone density and strength (109). Nevertheless, in patients with a history of kidney stones, the use of vitamin D supplements necessitates a more cautious approach and is accompanied by a rigorous risk/benefit assessment (77). In such cases, lower effective doses are recommended, and patients are advised that other precautions, such as increased water intake to promote the excretion of calcium and other salts, will not affect the incidence of incident kidney stones or hypercalcemia (19).

It is paramount that patients with a history of kidney stones who are using vitamin D supplements undergo regular monitoring of serum calcium and 25(OH)D levels, as well as urinary calcium excretion (77). These monitoring results can assist physicians in evaluating the patient’s response to vitamin D supplements and facilitate timely dosage adjustments to prevent potential complications. Serum 25(OH)D is a commonly utilized biomarker for assessing vitamin D status within the body. Regular monitoring of serum 25(OH)D levels is essential to ensure that vitamin D status remains within a healthy range. The normal range is generally considered to be 20–50 ng/mL (50–125 nmol/L), while levels above 100 ng/mL May indicate an overdose (83, 86). If a patient’s 25(OH)D level exceeds the desired range or urinary calcium excretion is elevated, it May be necessary to reduce the dose of vitamin D (Table 1).

**Table 1:** Vitamin D status and kidney stone prophylaxis.

Vitamin D status	Preventive measures
Deficiency (<20 ng/mL)	Increase sun exposure Increase intake of vitamin D-rich foods Vitamin D supplements Monitor serum 25(OH)D levels regularly Monitor 24,25(OH)₂D and 1,25(OH)₂D, if necessary
Moderate (20–100 ng/mL)	Maintain moderate sun exposure and dietary intake Avoid over-supplementation Regular health checkups
Excessive (>100 ng/mL)	Reduce use of vitamin D supplements Increase water intake to reduce urinary calcium concentration Closely monitor serum and urinary calcium levels

### 4.2 The role of lifestyle interventions in the prevention of kidney stones

The formation of kidney stones is a complex process influenced by multiple factors, with lifestyle habits playing a significant role. It is possible to significantly reduce the risk of kidney stones by modifying one’s lifestyle habits. Diet is an essential factor in the formation of kidney stones. Reducing salt intake reduces urinary calcium excretion, reducing the risk of specific kidney stones (110, 111). A diet high in protein, particularly animal protein, increases the production of uric acid and May result in the acidification of the urine, which contributes to stone formation (112–114). Reducing the consumption of red meat, poultry, and fish, accompanied by an increase in the intake of plant-based proteins, May reduce the risk of uric acid stones (115–117). Conversely, increased fruit and vegetable intake can provide a plentiful supply of potassium and magnesium. These minerals assist in reducing urinary calcium concentrations and lowering the risk of stone formation (118). Supplementing the standard diet with fresh lemon juice May prevent stone recurrence in patients with calcium oxalate kidney stones (119). Furthermore, the moderate consumption of foods rich in calcium (e.g., low-fat dairy products) May reduce the intestinal absorption of oxalates, thereby reducing the risk of calcium oxalate stones (110).

Increasing water intake is one of the most straightforward and productive methods for preventing kidney stones. It plays a pivotal role in preventing the formation of first-time kidney stones and reducing the risk of stone recurrence (118, 120–122). Adequate water intake dilutes stone-forming substances in the urine, reducing the likelihood of their deposition and crystallization. It is advised that fluid intake be increased to at least 2.5 liters per day to prevent the formation of stones (123–125). Furthermore, regular physical activity not only assists in maintaining a healthy weight but also contributes to calcium absorption and bone health by enhancing blood circulation and metabolism and increasing the body’s efficiency in utilizing vitamin D (126).

It is well-established that obesity represents a significant risk factor for the formation of kidney stones (127). Excessive adipose tissue is associated with increased uric acid production, increasing the risk of uric acid stones. Furthermore, obese individuals are more likely to engage in limited outdoor physical activity to avoid exposure to sunlight. They are more likely to consume diets low in vitamin D, which can result in vitamin D deficiency (128). Reducing the risk of kidney stones can be achieved by implementing a healthy diet and moderate exercise. Furthermore, weight management can also help improve insulin resistance and other metabolic abnormalities that influence kidney stone formation (118, 129).

While vitamin C is essential for health, excessive intake (primarily through supplements) May increase oxalic acid production, which increases the risk of oxalate stones (130). Both smoking and excessive alcohol consumption are associated with an increased risk of kidney stones (131, 132). Quitting smoking and limiting alcohol intake May improve overall health and reduce the risk of kidney stones (Table 2).

**Table 2:** Lifestyle interventions and kidney stone prevention.

Interventions	Preventive effects
Reducing salt intake	Reducing urinary calcium excretion and reducing stone risk
Control high protein diet	Reduce uric acid production to avoid acidification of urine and reduce stone risk
Increase intake of fruits and vegetables	Provide potassium and magnesium to reduce urinary calcium concentration and reduce stone risk
Increase calcium food intake	Decrease intestinal oxalate absorption, reduce calcium oxalate stone risk
Increase water intake	Dilute urine to reduce the chance of stone deposition and crystallization
Regular physical activity	Promote blood circulation and increase the efficiency of vitamin D utilization
Weight management	Improve insulin resistance, reduce uric acid production, reduce stone risk
Control vitamin C, smoking, and alcohol consumption	Avoid excessive vitamin C intake, stop smoking, and limit alcohol consumption to reduce stone risk

### 4.3 Importance of personalized medicine in vitamin D supplementation and kidney stone prevention

The concept of personalized medicine is predicated on tailoring treatment to the patient’s genetic background, lifestyle, and environmental factors. Genetics, lifestyle, and environmental factors significantly influence the individual’s need for and ability to metabolize vitamin D. Genetic factors, such as polymorphisms in the VDR gene, May affect the synthesis, distribution, and action of vitamin D. Furthermore, lifestyle factors, including diet, physical activity, and sun exposure, May also influence vitamin D status. Environmental factors, such as season and latitude, determine the intensity and duration of sunlight, which in turn affects the skin’s ability to synthesize vitamin D. These factors must be considered together when developing a vitamin D supplementation strategy to ensure individualized dosage and form. Furthermore, consideration should be given to the impact of hormones and pharmaceutical agents on vitamin D levels. Although serum 25(OH)D levels are relatively stable, they can be affected by various factors. These factors include thyroid hormones, anticonvulsants, choline, and orlistat. Furthermore, PTH and PTH-related peptides, prolactin, estradiol, testosterone, prostaglandins, and bisphosphonates, in addition to serum calcium and phosphorus, have been demonstrated to induce elevated serum 1,25(OH)_2_D levels. Conversely, corticosteroids, phospholipases, ketoconazole, heparin, and thiazides have been shown to reduce serum 1,25(OH)_2_D levels.

The patient’s overall health status and disease risk are crucial considerations when developing a prevention and treatment plan. For instance, patients with kidney stones May be advised to avoid high-dose vitamin D supplementation to reduce the risk of hypercalciuria. Conversely, patients at risk for osteoporosis May require a more aggressive vitamin D supplementation strategy to maintain bone health. Furthermore, the patient’s renal function, cardiovascular health, and metabolic status should be considered, as vitamin D metabolism occurs primarily in the liver and kidneys.

## 5 Limitations of current research and future research directions

### 5.1 Limitations of the current study

The current body of research on the relationship between vitamin D and kidney stones exhibits considerable inconsistency in its findings. This is evidenced by diverse and even contradictory results at times. This discrepancy May be attributed to variations in study design, sample selection, and experimental methodologies. For instance, some studies have indicated no association between vitamin D intake and the risk of kidney stones (17, 19, 92). In contrast, others have observed that excessive vitamin D intake May increase the risk of kidney stones (13, 95, 133). This inconsistency May also be related to the study populations’ genetic background, lifestyle, dietary habits, and dosage and form of vitamin D supplements. It is essential to control for confounding factors to establish causality. However, the complexity of the relationship between lifestyle and environmental factors and vitamin D status and kidney stone risk makes accurate measurement and control of these factors challenging.

The current study is somewhat limited in its scope. Previous studies have primarily focused on the correlation between vitamin D intake and the risk of kidney stones. However, relatively few studies have been conducted on the specific mechanisms of vitamin D metabolism and the differences in the effects of different forms of vitamin D (e.g., D2, D3) on kidney stones. Furthermore, the role of VDR gene polymorphisms in kidney stone formation and the variability among populations of different races and geographic regions have been understudied.

The limitations of sample size and duration of follow-up warrant consideration. Some studies’ relatively small sample sizes May not comprehensively represent the entire population. Additionally, the potential for bias in sample selection May result in inaccurate results. Additionally, the brief follow-up duration of certain studies May not permit an accurate evaluation of the long-term effects of long-term vitamin D intake on kidney stone risk. Longitudinal studies with extended follow-up periods are essential for understanding the dynamic relationship between vitamin D status and kidney stone formation.

The need for greater clarity regarding the dose–response relationship is evident. There is a lack of clarity regarding the dose–response relationship between vitamin D intake and the risk of kidney stones. The discrepancy between study results May be attributed to the differing vitamin D intake thresholds employed by different studies to define deficiency or excess. Additionally, no intervention studies have been conducted to assess the impact of adjusting vitamin D intake levels on preventing kidney stones. Such studies are crucial for developing effective prevention strategies and clinical guidelines. Furthermore, randomized controlled trials (RCTs) represent the gold standard for assessing the effectiveness of interventions. However, there May be ethical and practical challenges to implementing RCTs in studies of the effects of vitamin D supplementation on kidney stone risk.

### 5.2 Directions for future research

A comprehensive examination of the metabolic processes associated with vitamin D and their role in forming kidney stones. Future studies should aim to understand better the metabolic pathways of vitamin D in the body. In particular, they should investigate the conversion of vitamin D to its active form, 1,25(OH)_2_D, and its potential role in kidney stone formation. This process May affect intestinal calcium absorption and renal calcium excretion, which could contribute to the formation of kidney stones. Furthermore, research should concentrate on genetic variation in vitamin D metabolizing enzymes, such as polymorphisms in CYP27B1 and CYP24A1, critical enzymes in vitamin D metabolism. Determining how these polymorphisms affect an individual’s vitamin D metabolism and susceptibility to kidney stones is necessary.

Longitudinal studies with a large sample size and a long follow-up period are necessary to understand the relationship between vitamin D and kidney stones comprehensively. It is recommended that large-scale, long-term, population-based prospective studies be conducted to assess the long-term effects of different doses and forms of vitamin D intake on kidney stone risk. To gain a more comprehensive understanding of the relationship between vitamin D intake and kidney stone risk, these studies must include populations of different ages, sexes, ethnicities, and geographic locations. This will enable the development of more tailored recommendations for other populations. Despite the implementation challenges, future studies should consider conducting randomized controlled trials to assess the effectiveness of different vitamin D supplementation strategies in preventing kidney stones.

When studying the relationship between vitamin D metabolism and kidney stone risk, it is essential to consider the impact of individual differences and genetic factors. Future studies should examine the influence of an individual’s genetic background on this relationship. This encompasses the investigation of VDR gene polymorphisms and other genetic variants associated with calcium and phosphorus metabolism. These studies could lead to a more comprehensive understanding of the factors contributing to the higher risk of kidney stones observed in specific populations despite similar levels of vitamin D intake. This understanding could then inform the development of more targeted prevention strategies for high-risk individuals.

An integrated assessment of the combined effects of dietary and lifestyle factors is required. Future studies should integrate the impact of dietary patterns, nutritional intake, lifestyle factors such as physical activity and water intake, and environmental factors such as climate and altitude on vitamin D status and kidney stone risk. This multifactorial approach will facilitate the elucidation of how vitamin D intake and other lifestyle factors collectively contribute to the formation of kidney stones.

It is recommended that interdisciplinary collaboration and international collaborative research be employed. It is recommended that multidisciplinary collaboration, including experts in endocrinology, nutrition, nephrology, genetics, and epidemiology, be encouraged to facilitate a more comprehensive understanding of the relationship between vitamin D and kidney stones. Given the considerable diversity in genetic predispositions, dietary habits, and living environments among populations in different regions, international collaborative studies can facilitate the identification of generalizations and specificities in the vitamin D-kidney stone relationship across diverse populations. Future studies aim to assess the economic impact of different vitamin D supplementation strategies and examine how effective health policies can be developed to prevent vitamin D deficiency and overdose and reduce the incidence of kidney stones.

## 6 Conclusion

The relationship between vitamin D and kidney stones is intricate and multifaceted, encompassing a multitude of physiologic and metabolic pathways. Both excess and deficiency of vitamin D May increase the risk of kidney stones, necessitating the careful balancing of vitamin D intake. The relationship between vitamin D status and kidney stone risk varies across age, gender, and ethnicity, indicating the need for individualized assessment and management strategies to tailor vitamin D supplementation to the patient’s specific situation. Lifestyle modifications, such as dietary changes and increased physical activity, play a significant role in maintaining optimal vitamin D status and preventing the formation of kidney stones.

Future research is necessary to ascertain the causal relationship between vitamin D status and kidney stones, to evaluate the efficacy of various interventions, and to develop individualized treatment regimens. Further studies are required to ascertain the vitamin D requirements and kidney stone risk in specific patient populations. The results of the studies are likely to have significant social and economic implications, including the development of effective prevention strategies and the potential for healthcare cost savings. It is of the utmost importance that interdisciplinary collaboration be fostered and that the translation of research results into practical clinical and public health strategies be pursued with the utmost urgency.
